# Physical activity, cardiovascular health, quality of life and blood pressure control in hypertensive subjects: randomized clinical trial

**DOI:** 10.1186/s12955-018-1008-6

**Published:** 2018-09-14

**Authors:** Victoria Arija, Felipe Villalobos, Roser Pedret, Angels Vinuesa, Dolors Jovani, Gabriel Pascual, Josep Basora

**Affiliations:** 1 Unitat Suport a la Recerca Reus-Tarragona, Institut d’Investigació en Atenció Primária, IDIAP Jordi Gol (Barcelona), Camí de Riudoms 57, 43202 Reus, Spain; 2 Institut d’Investigació Sanitària Pere Virgili, Reus, Tarragona, Spain; 3 Faculty of Medicine and Health Sciences, Universitat Rovira i Virgili, Reus, Tarragona, Spain; 4 Primary Health Care Area, Reus, Tarragona, Spain; 5 Institut Català de la Salut, Generalitat de Catalunya, Barcelona, Spain

## Abstract

**Background:**

Physical activity (PA) promotes cardiovascular health and health related quality of life (HRQoL), although the effect of that on blood pressure (BP) control has rarely been studied in hypertensive subjects. Our aim was to evaluate the effectiveness of a PA intervention programme on cardiovascular disease (CVD) risk, HRQoL and BP control in hypertensive subjects.

**Methods:**

A randomized clinical trial, with a PA intervention programme of 9 months duration, comprising a walking group of 120 min/week, supervised, and with socio-cultural activities. Participants were 207 hypertensive subjects (68.2 years, 76.8% women). PA (IPAQ-s), diet, CVD risk, BP, BMI, smoking, and HRQoL (SF-36) were assessed at baseline and at the end of the intervention. Changes in CVD risk and in HRQoL during the intervention was calculated (end-baseline score). Multivariate models were applied.

**Results:**

In multivariate models, the PA intervention programme, with no modification of the diet, decreased CVD risk (− 1.19 points) and the systolic BP (− 8.68 mmHg), and increased some areas of HRQoL (4.45 to 14.62 points). An increase in the percentage of subjects with controlled BP was observed by the PA programme itself (OR 5.395 to 5.785 according to multivariate models), and by the changes during the intervention in the decrease in CVD risk (OR 0.609) and in the increase in the HRQoL in physical component summary (OR 1.041), role physical (OR 1.010), and bodily pain (OR 1.014), independently of controlled BP at baseline.

**Conclusions:**

This PA intervention programme improved cardiovascular health and HRQoL, and favoured BP control in primary care users with hypertension.

**Trial registration:**

Clinicaltrials.gov ID NCT02767739; Trial registered on May 5th, 2016. Retrospectively registered.

## Background

Hypertension is the most common chronic disease recognized as a major risk factor for other chronic diseases such as cardiovascular disease (CVD), type 2 diabetes mellitus (T2DM) and chronic kidney disease [1]. The prevalence of hypertension in developed countries varies from 30 to 45%, with this percentage increasing from 60 years of age [2]. In Spain, hypertension affects 42.6% of the adult population [3]. Its complications seriously increase the financial burden on the public health services [4]. In Spain causes around 5 million hospital admissions and contributing to approximately 40,000 cardiovascular deaths annually [3].

Hypertension is far from being controlled throughout the world, despite a large number of antihypertensive drugs being prescribed. The percentage of subjects with uncontrolled hypertension (≥140/90 mmHg) is high, ranging from 31.9–36.8% in adult population [2]. In a study conducted in Spain it is described that while the percentage of subjects who receive two or more antihypertensive drugs has increased in recent decades, prevalence of uncontrolled hypertension has not changed significantly [5]. Given this situation, in addition to pharmacological treatment, it seems important to implement strategies to promote healthy habits that help to avoid the main risk factors of arterial hypertension (i.e. sedentary lifestyle, stress, poor quality of life, unbalanced diet, smoking), and, at the same time, follow protocols that help to treat and control the hypertension [1].

The promotion of physical activity (PA) is one of the modifiable risk factors that has a beneficial effect on cardiovascular disease (CVD) risk. Several randomized clinical trials (RCT) with PA intervention programmes have reported a reduction in CVD risk in the adult population. In a recent meta-analysis of 26 RCTs with PA intervention programmes, a reduction in BP was reported in hypertensive subjects (− 8.3 mmHg, systolic blood pressure (SBP) and − 5.2 mmHg diastolic blood pressure (DBP)) [5], although none of the RTCs indicated the percentage of subjects with controlled hypertension in these studies.

In relation to HRQoL, various studies have addressed it relationship with hypertension. According to a meta-analysis of 20 observational studies, hypertensive subjects have poorer levels of HRQoL than non-hypertensive subjects [6]. This lower perception of HRQoL has been related to the frequent presence of comorbidities, with the side effects of antihypertensive drugs (headaches, dizziness, tinnitus, nausea, etc.) and, in particular, with the difficulty in being able to control BP through prescribed therapeutic procedures [7–9].

However, although there have been several studies into the effect of PA on HRQoL, results have been contradictory, and there have still been very few studies carried out with hypertensive subjects. We only found 2 RCTs, with Asian populations, that have studied this relationship, both of which found a positive effect of PA on HRQoL in hypertensive subjects [10, 11]. Another RCT conducted with Asian hypertensive subjects went one step further, and reported that the improvement in HRQoL (general health and perception of bodily pain), by itself, decreased SBP [10]. In addition, certain favourable characteristics in the designs of PA programmes aimed at improving HRQoL have been reported, such as group interventions, counselling [12–15], supervision [16] and those that included socio-cultural activities [17].

Given the importance of BP control, more studies would be needed to confirm this effect through the promotion of the PA and of the HRQoL. We believe that a PA programme that is designed to include the most favourable methodological characteristics will improve CV health and HRQoL, and these improvements will, in turn, favour BP control. Therefore, we aimed to evaluate the effectiveness of a PA intervention programme (group, supervised and with socio-cultural activities) on CVD risk, HRQoL and BP control in hypertensive subjects.

## Methods

### Study design

A RCT, with a PA intervention programme of 9 months duration, comprising a walking group of 120 min/week, supervised, and with socio-cultural activities.

### Study population

Five Primary Care Centres (PCCs) participated, four urban, in a city of 100,000 habitants (Reus, Catalonia, Spain) and one rural, near to the city.

Subjects were selected who had arterial hypertension (controlled or uncontrolled) diagnosed more than one year earlier, with these inclusion criteria: adult, primary health-care user, signed informed consent, and with no exclusion criteria such as having suffered an event of ischaemic heart disease (< 6 months), severe acute or inter-recurrent acute disease requiring hospital admission or medical rest, an outbreak of osteoarthritis that would limit ambulation, pulmonary or heart disease with dyspnoea at small or moderate effort.

The sample size was recalculated according to the results of the main dependent variables of the study, apart from: alpha risk of 0.05, beta risk < 0.2, unilateral contrast, 3:1 ratio for intervention group (IG)/control group (CG). For CVD risk 152/50 subjects (SD: 3.7 points; difference: 1.5 units; average and difference between GI and GC, respectively, Table 2). For physical component summary 158/52 subjects (SD: 8.05 points (8.3 and 7.8, Table 3), difference: 3.2 points (46.1–49.3, Table 3). For BP controlled 154/50 subjects (0,40% GC and 0.60% GI (20% increase, Table 2). (Version 7.12; Granmo software; IMIM Hospital del Mar, Barcelona, Spain).

### Procedure and intervention

Health-care professionals (physicians and nurses) of the participating PCCs invited hypertensive subjects to participate in the study over 6 months prior to the intervention. Volunteers who agreed to participate were directed to the nurse responsible for the study in each PCC for an assessment of their eligibility criteria. Participants were randomized individually by the research coordinator through a computer program into the IG or the CG at a 3:1 ratio.

The PA intervention programme consisted of supervised group walking sessions (396 METs/min/week over 120 min, in 2 sessions of 60 min), according to international recommendations on PA [18], and with monthly socio-cultural activities. A nurse and a PA specialist accompanied the participants in all the activities (supervisors), which were pre-set. The walks consisted of circuits of about five kilometres around the city. Socio-cultural activities included visits to museums, libraries, cultural exhibitions, tourist attractions and dance classes. Group sizes ranged from 15 to 30 participants.

CG received standard clinical care by the health-care professionals [19].

### Variables

#### Outcomes measured at baseline

##### Clinical history and socio-demographic characteristics

Information on age, gender, social class, smoking and comorbidities (such as T2DM, hyperlipidaemia, overweight, obesity, depression, anxiety and osteoporosis) was obtained from face-to-face interviews. Social class was adapted from the British Registrar General classification which yields three class categories: high (class I-II), middle (class IIIN-IIIM) and lower (class IV-V) [20]_._

#### Outcomes measured at baseline and at the end of the PA intervention programme

##### Physical activity

Levels of PA were measured using the short version of the International Physical Activity Questionnaire (IPAQ-s), validated for the Catalan population [21]. Intensity (walking, moderate, or vigorous), frequency and duration of PA were registered. The min/week of each PA intensity was calculated. The metabolic equivalent of task (METs)/min/week were obtained by multiplying the average energy expenditure by min/week for each PA intensity (3.3 MET for walking, 4.0 MET for moderate intensity, and 8.0 MET for vigorous intensity). The results of each category of PA (walking + moderate intensity + vigorous intensity) were added to obtain the total physical activity in METs/min/week [22].

##### Frequency of food consumption

Food consumption was assessed using a validated food frequency questionnaire containing 45 items [23]. Through interviews, a nurse recorded the occasions per week or month that rations were consumed, and the daily average was calculated from that. To calculate g/day of each food item, daily rations were multiplied by grams of each item consumed relative to reference data of food consumption evaluated in the same population [24]. Food was grouped as follows: dairy products (milk, yogurt, dairy desserts, cheese); meat/fish/eggs (red, white, processed meat and cold meat, lean, fatty fish and shellfish); cereals (rice, pasta, bread, legumes and potatoes, pastries, biscuits, breakfast cereals); fruit/vegetables (salad, tomatoes, vegetables side dish, courgettes, mushrooms; green beans, chards, spinach, fresh fruit and canned fruit); nuts; sugary beverages; and alcoholic beverages.

##### Cardiovascular disease risk assessment

The *“Registre Gironí del Cor”* (REGICOR) scale was recorded from the computerized clinical histories. This scale values the overall CVD risk, based on the Framingham criteria standardized for the Spanish population. The scale includes gender, age, diabetes (no, yes), smoking (no, yes), SBP, DBP and serum cholesterol levels [25]**.**

BP was measured with a manual sphygmomanometer with the participants resting for at least five minutes. Three recordings were taken and the average of the second and third readings was used in the statistical analyses. Having controlled BP was considered when it met the European Society of Cardiology criteria [26] with SBP and DBP values of < 140/90 mmHg, respectively; and uncontrolled when any of these conditions were breached. Percentage of subjects with controlled BP and the percentage change during intervention from controlled to uncontrolled and from uncontrolled to controlled (end-baseline) was calculated.

Weight (kg), height (cm) and waist circumference (cm) were measured and body mass index [BMI as kg/m^2^] was calculated^.^

### Health-related quality of life

This was evaluated using the Spanish version of the Short Form Health Survey (SF-36), estimating 2 components. The physical component summary comprises 4 domains (physical function, role physical, bodily pain and general health) and the other 4 the mental component summary (vitality, social function, role emotional and mental health). Each variable scores from 0 to 100, with high scores indicating a better quality of life [27]. Change in HRQoL during intervention (end - baseline values) was calculated.

### Data analysis

All categorical variables were described as percentages, while means and standard deviations were reported for continuous variables. The χ^2^ test was used to compare categorical variables in different groups, and Student’s *t*-test to compare continuous variables.

Multiple linear regression models were applied to assess the effect of the intervention (no, yes) (independent variable) on the CV health (score) and on the components or domains of the HRQoL (score) (dependent variables). The following baseline co-variables were included: age (years), gender (men, women), social class (DUMMY variables were created: social class low, reference versus middle and high), PCC (DUMMY variables were created: PCC1, reference versus PCC2, PCC3, PCC4, PCC5), IMC (Kg/m^2^), smoking (no, yes), comorbidity (number of chronic diseases), controlled BP (no, yes) and dependent variable of each model at baseline.

Logistic regression models were applied to assess the effect of the change (end-baseline score) during the intervention in CVD risk and in physical and mental components or domains of the HRQoL, as independent variables, on BP control (no, yes) (dependent variable). These models were adjusted for the same variables as the previous multiple linear regression models, except for the dependent variable of each model at baseline.

Statistical significance was set at *p* value < 0.05. The statistical software SPSS for Windows Version 22.0 (SPSS Statistics 22.0) was used throughout.

## Results

**Fig. 1:**
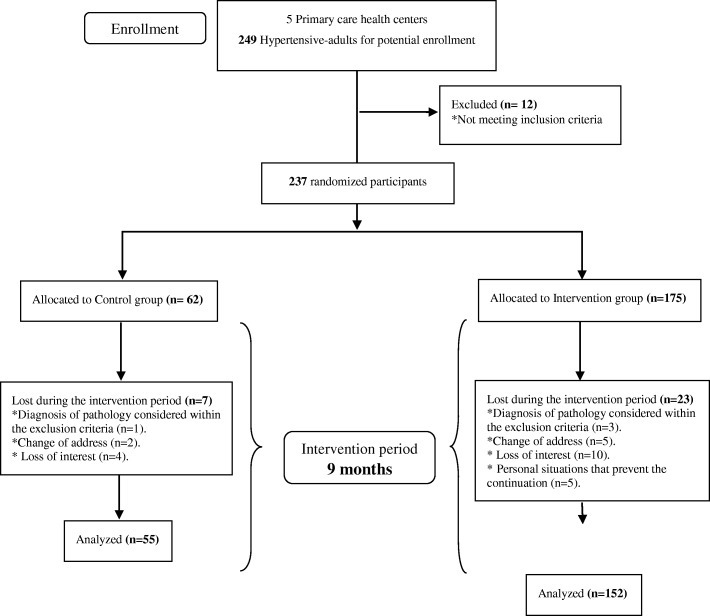
Study Flow Diagram

There were 237 participants (IG = 175; CG = 62). During the intervention, 23 subjects from the IG and 7 from the CG dropped-out, therefore 207 participants completed the study (IG = 152; CG = 55) (Fig. 1). The participants who dropped out had the same characteristics as those who completed the study in terms of age, gender, social class and comorbidity (*p* > 0.05).

There were no significant differences in the socio-demographic characteristics, smoking, comorbidities, food consumption, or in PA at baseline. Total PA significantly increased at the end of the intervention in the IG, whereas it decreased in the CG (Table 1).

**Table 1:** Socio-demographic characteristic, health status, food consumption and physical activity during physical activity intervention program in hypertensive subjects

	Baseline		*p*	End		*p*
	Control group (*n* = 55)	Intervention group (*n* = 152)		Control group (n = 55)	Intervention group (n = 152)	
Age (years)^a^	70.1 (9.3)	67.4 (6.6)	0.057			
Women (%)	72.7	76.3	0.589			
Social Class						
High Class I-II (%)	21.8	19.1	0.070			
Middle Class III_N_-III_M_ (%)	70.9	53.3				
Lower Class IV-V (%)	7.3	27.6				
Chronic diseases						
T2DM (%)	23.6	25.7	0.857			
Overweight (%)	34.5	41.4	0.423			
Obesity (%)	60.0	48.0	0.157			
Dyslipidemia (%)	60.0	56.6	0.751			
Depression (%)	10.9	15.1	0.505			
Anxiety (%)	25.5	12.5	0.320			
Osteoporosis (%)	6.0	8.2	0.060			
Comorbid diseases (n)^a^	2.2 (1.0)	2.2 (1.0)	0.703			
Food consumption^a^						
Dairy Products (g/day)^a^	348.7 (161.3)	316.7 (150.9)	0.214	381.6 (188.5)	335.0 (173.3)	0.117
Meat/Fish/ Eggs (g/day)^a^	147.9 (46.7)	142.4 (57.8)	0.542	145.1 (53.0)	152.0 (66.1)	0.503
Cereals (g/day)^a^	155.9 (59.1)	137.8 (49.8)	0.068	154.6 (68.6)	134.0 (49.3)	0.813
Fruits/ Vegetables (g/day)^a^	329.3 (137.2)	281.6 (122.4)	0.260	318.8 (157.9)	298.3 (140.6)	0.402
Sugary beverages (g/day)^a^	16.6 (43.7)	22.0 (56.0)	0.538	22.9 (59.6)	21.9 (46.9)	0.908
Physical activity intensity^a^						
Walking (METs/min/week)^a^	1459.0 (1431.9)	1363.0 (1997.8)	0.556	1565.0 (1997.8)	1912.0 (1545.4)	0.670
Moderate (METs/min/week)^a^	484.0 (1230.8)	481.0(1210.1)	0.619	279.0 (760.5)	930.0 (1633.6)	0.004
Vigorous (METs/min/week)^a^	164.0 (169.6)	161.0 (820.4)	0.165	161.0 (160.3)	208.4 (868.2)	0.077
Total (METs/min/week)^a^	2107.0 (2156.5)	1995.3 (2112.7)	0.742	2005.2 (1989.2)	3050.0 (2466.4)	0.002

*T2DM* Type 2 diabetes mellitus, *METs* Metabolic equivalents task

^a^Values expressed as mean and standard deviation (SD)

There were no significant differences in CVD risk observed between groups at baseline (Table 2). At the end of the intervention the IG, compared to the CG, decreased CVD risk more than the CG and the SBP and increased the percentage of subjects with controlled BP and tended toward weight reduction (*p* = 0.059) and BMI (*p* = 0.062).

**Table 2:** Cardiovascular health during physical activity intervention program in hypertensive subjects

	Baseline		*p*	End		*p*
	Control group (*n* = 55)	Intervention group (*n* = 152)		Control group (*n* = 55)	Intervention group (*n* = 152)	
CVD risk (score)^a^	5.5 (4.0)	4.8 (3.4)	0.216	6.1 (4.3)	4.6 (3.2)	0.032
Weight (Kg)^a^	79.7 (14.7)	76.1 (14.0)	0.117	79.3 (15.1)	75.0 (14.4)	0.059
BMI (Kg/m^2^)^a^	31.6 (4.4)	30.5 (4.3)	0.128	31.4 (4.6)	30.0 (4.4)	0.062
Smoking (%)	3.6	5.9	0.731	3.6	4.6	0.556
Systolic BP (mmHg)^a^	135.4 (15.0)	134.5 (16.1)	0.690	139.3 (20.1)	131.8 (13.7)	0.002
Diastolic BP (mmHg)^a^	75.9 (10.0)	76.7 (9.8)	0.554	74.9 (12.0)	74.8 (8.3)	0.953
Controlled BP (%)	39.6	42.8	0.751	32.4	51.3	0.015
Change in control of BP • From uncontrolled to controlled (%)				12.3	20.4	0.042
• From controlled to uncontrolled (%)				23.1	13.6	

*CVD* cardiovascular disease, *BMI* Body mass index. *BP* blood pressure

Change in control of BP (end-baseline)

^a^Values expressed as mean and standard deviation (SD)

Table 3 shows differences in some of the domains of HRQoL at baseline. For this reason we calculated the variable change during the PA intervention (end-baseline) in each domain of the HRQoL. Positive changes were observed in the IG in the domains of physical function (*p* = 0.045), general health (*p* = 0.034) and vitality (*p* = 0.031), compared to the CG.

**Table 3:** HRQoL during physical activity intervention program in the hypertensive subjects

	Baseline		*p*	End		*p*	Change during physical activity intervention program (End - Baseline)		*p*
	Control group (*n* = 55)	Intervention group (*n* = 152)		Control group (n = 55)	Intervention group (*n* = 152)		Control group (*n* = 55)	Intervention group (*n* = 152)	
Physical component									
Summary (score)	42.1 (9.6)	46.1 (7.8)	0.001	43.3 (8.2)	49.3 (8.3)	0.001	0.7 (8.3)	3.2 (7.8)	0.307
Physical function (score)	68.3 (26.7)	78.8 (17.6)	0.001	69.0 (24.0)	84.6 (16.4)	0.001	0.1 (21.9)	5.9 (18.2)	0.045
Role physical (score)	66.1 (42.7)	77.4 (37.0)	0.047	69.5 (42.8)	83.0 (33.0)	0.010	2.6 (51.1)	5.7 (40.0)	0.637
Bodily pain (score)	56.7 (27.3)	68.4 (26.2)	0.003	60.5 (28.8)	72.3 (26.9)	0.003	2.7 (33.4)	4.4 (27.8)	0.698
General health (score)	55.7 (17.0)	57.1 (16.8)	0.573	54.6 (19.6)	60.9 (18.4)	0.022	−1.0 (15.5)	3.7 (15.5)	0.034
Mental component									
Summary (score)	48.6 (13.7)	46.8 (10.7)	0.305	47.2 (13.5)	47.6 (11.7)	0.809	−0.7 (11.6)	0.7 (12.7)	0.423
Vitality (score)	56.0 (24.6)	63.7 (19.5)	0.013	55.5 (21.6)	65.2 (21.3)	0.003	−1.1 (16.6)	1.4 (18.4)	0.031
Social function (score)	76.5 (29.7)	82.0 (19.7)	0.105	77.2 (27.6)	83.5 (22.3)	0.068	0.5 (23.9)	1.6 (23.8)	0.766
Role emotional (score)	80.5 (35.3)	75.3 (36.3)	0.325	77.8 (39.2)	79.4 (35.4)	0.777	−2.5 (40.0)	3.9 (46.6)	0.327
Mental health (score)	68.6 (25.4)	68.1 (19.0)	0.858	65.8 (22.8)	70.7 (20.2)	0.131	−2.5 (18.2)	2.4 (18.5)	0.066

Values are expressed as mean and standard deviation (SD)

Table 4 shows the effects of the PA intervention programme on CV health and on HRQoL in multiple linear regression models. This programme reduced by − 1.19 (*p* = 0.024) the score of the CVD risk, and by − 8.68 mmHg the level of the SBP (*p* = 0.001), independently of the baseline values. A favourable effect of the intervention on the HRQoL was observed in the physical component summary (5.00 points, *p* = 0.036), physical function (14.62 points, *p* = 0.001), role physical (13.59 points, *p* = 0.020), bodily pain (10.84 points, *p* = 0.016), general health (6.56 points, *p* = 0.011), and vitality (4.45 points, *p* = 0.050), independently of the baseline levels of each component or domain.

**Table 4:** Effects of physical activity intervention program on cardiovascular health and on HRQoL in hypertensive subjects

Cardiovascular health				
	β	SE	*p*	
Model 1. CVD risk (score)				
PA intervention (no, yes)	−1.19	0.52	0.024	R^2^_C_ × 100 = 49.8%; F_18,219_ = 13.20; *p* = 0.001
Gender (men, women)	− 1.07	0.49	0.032	
Controlled BP at Baseline (no, yes)	−1.01	0.45	0.018	
Baseline cardiovascular risk (score)	0.64	0.06	0.001	
Model 2. Systolic blood pressure (mmHg)				
PA intervention (no, yes)	−8.68	2.54	0.001	R^2^_C_ × 100 = 21.7%; F_18,219_ = 5.38; *p* = 0.001
Smoking (no, yes)	15.44	4.57	0.001	
Baseline systolic blood pressure (mmHg)	0.22	0.10	0.036	
HRQoL				
	β	SE	*p*	
Physical component of HRQoL				
Model 3. Summary (score)				
PA intervention (no, yes)	5.00	1.41	0.001	R^2^_C_ × 100 = 37.5%; F_18,219_ = 8.55; *p* = 0.001
Comorbidity (n)	−1.22	0.54	0.001	
Summary at baseline (score)	0.44	0.67	0.001	
Model 4. Physical function (score)				
PA intervention (no, yes)	14.62	2.80	0.001	R^2^_C_ × 100 = 47.1%; F_18,219_ = 13.14; *p* = 0.001
Physical function at baseline (score)	0.42	0.05	0.001	
Model 5. Role physical (score)				
PA intervention (no, yes)	13.59	2.80	0.001	R^2^_C_ × 100 = 28.2%; F_19,217_ = 5.83; *p* = 0.001
Gender (men, women)	−18.64	6.48	0.001	
Comorbidity (n)	−7.40	2.29	0.001	
Role physical at baseline (score)	0.27	0.06	0.001	
Model 6. Bodily pain (score)				
PA intervention (no, yes)	10.84	4.48	0.016	R^2^_C_ × 100 = 28.7%; F_18,219_ = 5.83 *p* = 0.001
Bodily pain at baseline (score)	0.43	0.66	0.001	
Model 7. General health (score)				
PA intervention (no, yes)	6.56	2.54	0.011	R^2^_C_ X 100 = 46.5%; F_18,220_ = 12.82 *p* = 0.001
Comorbidity (n)	−2.40	0.06	0.021	
General health at baseline (score)	0.65	0.06	0.001	
Mental component of HRQoL				
Model 8. Vitality (score)				
PA intervention (no, yes)	4.45	2.93	0.050	R^2^_C_ X 100 = 49.6%; F_18,220_ = 14.62 *p* = 0.001
Vitality at baseline (score)	0.64	0.05	0.001	

PA: Physical activity. CVD: cardiovascular disease. Multiple Linear Regression models adjusted for age (years), gender (men = 0, women = 1), social class (DUMMY variables, 0 = reference), 5 Primary Care Centers (DUMMY variables, 0 = reference), IMC (kg/m^2^), smoking (no = 0, yes = 1), controlled BP at baseline (no = 0, yes = 1), comorbidity (n = number of chronic disease), dependent variable of each model at baseline. Only significant models are shown

Table 5 shows a positive effect on percentage of subjects with controlled BP at the end of the intervention produced by the change in CVD risk during intervention (OR 0.609), and by changes in HRQoL in the physical component summary (OR: 1.041), in the role physical (OR: 1.010), and in bodily pain (OR: 1.014), in logistic regression models. All these associations are independent of the PA intervention programme and of having controlled BP at baseline. These results highlight the powerful effect of the PA intervention programme on all models (OR 5.395 to 5.785).

**Table 5:** Relation between the change in the cardiovascular health and the change in the HRQoL during physical activity intervention program and the control of the blood pressure at end of intervention in hypertensive subjects

	Control of the BP (no, yes)			
	Exp (β)	95% CI	*p*	
Model 1. Cardiovascular health				
Change in CVD risk (end-baseline score)	0.609	0.463–0.800	0.001	R^2^ Nagelkerke ×100 = 44.4,
PA intervention (no, yes)	5.395	1.827–15.934	0.002	χ^2^_78 239_ = 60.95
Controlled BP at baseline (no, yes)	6.603	2.474–17.627	0.001	*p* = 0.001
Model 2. Physical component summary of HRQoL				
Change in physical component summary (end-baseline score)	1.041	1.002–1.082	0.040	R^2^ Nagelkerke x 100 = 29.5,
PA intervention (no, yes)	5.402	2.377–12.275	0.001	χ^2^_32 239_ = 49.17
Controlled BP at baseline (no, yes)	2.853	1.433–5.680	0.003	*p* = 0.001
Model 3. Role physical of HRQoL				
Change in role physical (end-baseline score)	1.010	1.001–1.018	0.025	R^2^ Nagelkerke x 100 = 29.8,
PA intervention (no, yes)	5.785	2.524–13.257	0.001	χ^2^_32 239_ = 49.76
Controlled BP at baseline (no, yes)	2.581	1.304–5.110	0.007	*p* = 0.001
Model 4. Bodily pain of HRQoL				
Change of bodily pain (end-baseline score)	1.014	1.002–1.027	0.024	R^2^ Nagelkerke x 100 = 29.5,
PA intervention (no, yes)	5.635	2.469–12.863	0.001	χ^2^_34 239_ = 48.95
Controlled BP at baseline (no, yes)	2.672	1.347–5.302	0.005	*p* = 0.001

Blood pressure (BP). PA: Physical activity. CVD: cardiovascular. BP = blood pressure. Logistic Regression model adjusted for physical activity (PA) intervention (no = 0, yes = 1), age (years), gender (men = 0, women = 1), Social class (DUMMY variables, 0 = reference), 5 Primary Care Centers (DUMMY variables, 0 = reference), IMC (Kg/m^2^), smoking (no = 0, yes = 1), comorbidity (n = number of chronic disease) and controlled BP at baseline (no = 0, yes = 1). Only significant models are shown

## Discussion

This PA intervention programme of 9 months duration, with a supervised walking group of 120 min/week and socio-cultural activities was aimed at hypertensive primary care users. The programme increased the level of PA, and improved CV health (decreased CVD risk and SBP) and some components or domains of the HRQoL in the IG. In addition, these favourable changes produced during the intervention in CVD risk and in HRQoL (physical components or domains physical component summary, role physical and bodily pain) increased the percentage of subjects with controlled BP. Our results estimated in multivariate models and without modification of dietary habit, contribute to the scarce knowledge of the effect of PA on the improvement of HRQoL in hypertensive subjects, and provide evidence of their effect on BP control, showing the importance of establishing strategies for health promotion and HRQoL among subjects with arterial hypertension.

The results of this study are supported by the strong evidence of an RCT, which used validated questionnaires [21, 23, 27] and controlled the related factors (age, gender, lifestyles and comorbidity) through multivariable statistical techniques. A random assignment was performed at a 3:1 ratio, higher for the IG, to favour the potential benefits of PA on health (according to the published literature) for a greater number of participants. The PA intervention programme included the PA recommendations proposed by international organizations [18] and methodological characteristics of previous PA intervention programmes that showed greater benefits on health: the walking groups [28], socio-cultural activities [29, 30] or supervision by health care professionals and/or PA specialists [31]. However, a limitation of the study is the fact that the CG did not take part in any activity as an alternative to the intervention.

During the intervention, no food advice was given to the participants so that they would not modify their food consumption and results could therefore be interpreted irrespective of diet. This control is rarely included in the reviewed RCTs, despite diet being a very related factor for BP.

Our results show that total PA significantly increased by 1054.7 (±2926.4) METs/min/week in the IG, between baseline and the end of the intervention, whereas it decreased by 101.8 (±2320.7) METs/min/week in the CG. In the IG, approximately 35% of the PA corresponds to the walking in the intervention programme and 65% to other activities outside the programme. The data clearly indicates the effectiveness of this programme in promoting different leisure-time activities. Some authors have linked these favourable results to some of the characteristics of the programme, such as being supervised, the walking in groups and the socio-cultural activities, all of which probably encouraged these improvements [28–31].

### PA and CV health

The PA intervention programme showed a reduction in the overall CVD risk and in some of its components, principally the SBP levels (from 134.5 to 131.8 mmHg, *p* = 0.002) and in the percentage of subjects with controlled BP. I-n addition to a trend, no significant, in the decrease in body weight and BMI. In the multivariate models, CVD risk reduction was confirmed (− 1.19 points, *p* = 0.024), and the decrease in SBP was even higher in the multivariate analysis (− 8.68 mmHg, *p* = 0.001) compared to the bi-variant analysis.

Other RCTs conducted in developed countries with hypertensive subjects yielded results consistent with ours. A recent meta-analysis of 26 RCTs with PA intervention programmes of 45–180 min/week and of 6–56 weeks duration observed a significant reduction in SPB (− 8.3 mmHg) [5]. However, none of those studies assessed weight or BMI. There is strong evidence in the general population that physical activity is a determining factor in the decrease and maintenance of a healthy weight, with greater benefits being observed when it is above 150 min/week [32]. A RCT that included a PA intervention programme of 165–220 min/week with hypertensive subjects observed a significant reduction in body weight (− 1.8 kg) and BMI (− 0.6 kg/m2), results that were better than in the present study (− 1.1 kg and − 0.05 kg/m2, respectively) [33].

The increase in the percentage of subjects with controlled BP in the IG at the end of the intervention, rising from 40.8 to 49.3%, contrasts with a reduction from 43.6 to 36.4% in the CG (*p* = 0.015). The same trend is observed in the change from uncontrolled to controlled BP, since it is significantly higher in the IG (20.4%) than the CG (12.3%) (*p* = 0.042). There is a paucity of knowledge about the effect of PA on controlled BP. Most of the reviewed studies value the effect of PA on the levels of BP. As an exception, an study observed an increase from 11.8–19.7% of hypertensive subjects who were able to control BP as a result of an intervention programme based on advice on diet and exercise and of the adherence to antihypertensive treatment over 6 months [34].

The mechanisms involved in the reduction of SBP through physical activity might be related to a cardiac remodelling [35], and a decrease in peripheral vascular resistance, which might be due to neurohormonal and structural responses with reductions in sympathetic nerve activity and increases in arterial lumen diameters [36]. Other mechanisms proposed are that PA increases endothelial function [37] and decreases oxidative stress, the inflammation syndrome [38], the renin-angiotensin system [39], parasympathetic activity and renal function [39].

### PA and HRQoL

The benefits of the PA intervention programme on HRQoL is observed in some domains, mainly in the physical area, such as physical function, general health and vitality. These results are mostly consistent with the results from multivariate analyses adjusted for confounding factors, where a positive effect was also observed in physical function (14.62 points), general health (6.56 points) and vitality (4.45 points) in addition in the component summary (5.00 points), role physical (13.59 points) and bodily pain (10.84 points).

RCTs that have evaluated the relationship between PA and HRQoL have found contradictory results and appear related to the characteristics of the PA programme. While studies based on PA advice [12–15] or supervised aerobic PA programmes [16, 17, 40] reported a positive relationship, the studies that were not supervised, with high levels of aerobic PA [41] or with high intensity [42, 43] did not reported any benefit on HRQoL.

However, none of the previous RCTs have been conducted with hypertensive subjects. To our knowledge, only two studies have reported this type of population, both from Asia. One of them carried out a supervised PA intervention programme of 150 min/week and of 10 weeks duration. It observed an increase in HRQOL, with values a little higher than the present study in the physical domains between CG and IG: physical function (86.3 vs 92.3 points), role physical (76.7 vs 83.3 points) and bodily pain (73.3 vs 83.6 points). Moreover, it reported benefits in more domains than we did in the mental component: social function (74.3 vs 83.3 points), role emotional (60.0 vs 84.5), and vitality (60.7 vs 72.2) [10]. The other RCT was based on a 12-month Tai Chi Training Programme, with 180 min/week of PA, and reported benefits in similar domains of HRQoL, although with higher values than we did: role physical (83.27 vs 94.58 points), bodily pain (79.48 vs 90.83 points) and vitality (74.40 vs 84.17 points) [11].

At the physiological level, the sense of well-being associated with the practice of PA might be related to the release of neurotransmitters, such as serotonin, dopamine or noradrenaline, which act at the brain level, increasing the feeling of well-being and also through the inhibition of nerve fibres that transmit pain, by producing a certain degree of anaesthesia [44]. On the other hand, it seems that the PA programmes supervised by health professionals, such as the present study, have had favourable effects on the HRQoL [16, 17, 40]. It has also been observed that group activities, such as walking or carrying out socio-cultural activities, as our programme did, improve the well-being and mental health of the individual through the creation and consolidation of social support networks, which favour the development of bonds between individuals through the exchange of feelings, thoughts and experiences during these activities [45].

### Changes during PA intervention programme and BP control

The main objective in the treatment of arterial hypertension is to maintain levels within normality, although it remains difficult to achieve [1]. Taking this into account, the present study evaluated the effect of the changes (end - baseline) produced during intervention in CVD risk and in HRQoL on the percentage of subjects with controlled BP, through logistic regression models, adjusted for PA programme intervention, and for control of BP at baseline, among other variables.

There was a decrease in CVD risk during the intervention. It must be taken into account that the negative values in this variable are favourable, since they reduce CVD risk, therefore the values of OR < 1 (0.609) would be enhancers of the effect of the dependent variable, that is to say, that enhance the probability of having the controlled BP. For the interpretation of a OR < 1, it is better to calculate the inverse value (1/0.609 = 1.64), and interpret the decrease of 1 unit of CVD risk during the intervention as increasing 1.64 times the probability of having the controlled BP. The change or increase during intervention in physical component summary, role physical, and bodily pain of the HRQoL had a direct positive effect on BP control (OR: 1.041; 1.010 and OR: 1.014 respectively). These probabilities, although modest, are important, especially in the role physical of HRQoL, which changed during the intervention by 5.7 units (Table 3).

It is important to highlight that in the previous logistic regression models the variable that caused the greatest BP control was the PA intervention programme. The IG increased between 5.395 and 5.785 times the probability of having controlled BP, with respect to CG, independent of the benefit produced by the decrease in CVD risk and by the increase of some areas of the HRQoL, the relationship being adjusted by controlled BP at baseline and by the other confounding variables.

Although the mechanisms involved in the described relation between improvement of the HRQoL and BP control are not clear, this might be due to the fact that there is an association between HRQoL and stress and anxiety and better perception of HRQoL reduces the levels of stress and anxiety [46, 47]. These factors are linked to an increase in the sympathetic nerve activity [48, 49], which causes the high levels of BP [50, 51]. In addition, the improvement in HRQoL favours the acquisition of healthy behaviours, such as regular PA, a healthy diet, self-care and better support for adherence to anti/hypertension treatments [52].

Similar results were observed in an RCT carried out with the Asian hypertensive subjects who took part in a supervised PA programme, of 150 min/week of aerobic PA, mentioned above. The improvement during the intervention in the general health and the bodily pain of the HRQoL directly correlated with a decrease in SBP (*r* = 0.55, *p* = 0.030; *r* = 0.53, *p* = 0.040, respectively) [10].

These positive relationships between the completion of the programme, the decrease in CVD risk and the increase in the quality of life over BP control would support the benefit of promoting healthy lifestyles and quality of life as adjuvant actions for the treatment of the BP.

## Conclusion

The supervised PA intervention programme of 9-month duration, with a walking group of 120 min / week and with sociocultural activities, increased physical activity, reduced the CVD risk and systolic BP, and increased the HRQoL scores in the physical component summary and its domains and in the vitality of the mental component. In addition, the PA intervention programme itself, together with the reduction of the CVD risk during the intervention and the improvement in some areas of the HRQoL, such as the physical component summary, the role physical and bodily pain scores increased the percentage of hypertensive subjects with controlled BP at the end of the study.

## Abbreviations

BMI: Body mass index
BP: Blood pressure
CG: Control croup
CVD: Cardiovascular disease
DBP: Diastolic blood pressure
HRQoL: Health related q of life
IG: Intervention group
IPAQ-s: Short version of the international physical activity questionnaire
MET: Metabolic equivalent of task
PA: Physical activity
PCC: Primary care center
RCT: Randomized control trial
SBP: Systolic blood pressure
